# The effect of ketamine versus tramadol on prophylactic post-spinal shivering in those patients undergoing orthopedic surgery: a prospective cohort study design, 2020

**DOI:** 10.1186/s12871-022-01906-z

**Published:** 2022-11-24

**Authors:** Ashebir Debalike Gemechu, Tsegaye Demeke Gebremedhin, Andualem Assefa Andebiku, Fithamlak Solomon, Abebe Sorsa

**Affiliations:** 1 School of Anaesthesia, College of Health Science and Medicine, Wolaita Sodo University, P.O.BOX: 138, Wolaita Sodo, Ethiopia; 2 School of Medical Laboratory, College of Health Science and Medicine, Wolaita Sodo University, Wolaita Sodo, Ethiopia; 3 School of Public Health, College of Health Science and Medicine, Wolaita Sodo University, Wolaita Sodo, Ethiopia

**Keywords:** Ketamine, Tramadol, Orthopedic surgery, Post-spinal shivering, Spinal anesthesia

## Abstract

**Background:**

Post-spinal shivering is a common complication after spinal anesthesia with a high incidence among orthopedic patients. Untreated shivering may predispose to exacerbation of wound pain, increased metabolic demand, oxygen consumption, and hemostatic dysfunction. Various studies have been done on the effectiveness of preventing post-spinal shivering using ketamine and other drugs. However, little information on better prophylactic agents in terms of effectiveness and availability. Therefore, this study was intended to compare 0.25 mg/kg of Ketamine (K) versus 0.5 mg/kg of Tramadol (T) for the prevention of post-spinal shivering.

**Method:**

A prospective cohort study design was employed on 516 patients undergoing orthopedic surgery under spinal anesthesia, and they were selected by a consecutive sampling technique. Patients were divided into two groups based on the anesthetist in charge. Patients who received an intravenous prophylactic dose of Ketamine before spinal anesthesia are called Ketamine groups and patients who received Tramadol are called Tramadol groups (control). The severity and incidence of shivering, blood pressure, heart rate, and axillary body temperature were measured and recorded for one hour at 10-min intervals during the intraoperative period. Descriptive statistics, chi-square, independent t-test, and multivariable logistic regression were used. Significance was declared at a *p*-value lower than 0.05.

**Results:**

The overall incidence of post-spinal shivering was 187 (36.2%), of which it was 74 (28.7%) on ketamine and 113 (43.8%) on tramadol with a *p*-value of 0.001. The incidence of nausea and vomiting was 157 (60.9%) on tramadol and 8 (3.1%) on ketamine, with a *p*-value of 0.001. Patients aged 18–35 years (AOR 0.08 (0.02, 0.27), 36–55 years (AOR 0.24, 0.07, 0.81), and those patients with a prolonged duration of surgery (AOR 1.47 (1.37–1.58)) were more likely to experience post-spinal shivering. And Low-dose ketamine has a protective effect against developing post-spinal shivering with an AOR of 0.427 (0.28–0.63).

**Conclusion:**

Low-dose ketamine is more effective in reducing the incidence and severity of shivering after spinal anesthesia. Therefore, we recommend using low-dose ketamine to be effective as a prophylactic for post-spinal shivering in those patients undergoing orthopedic surgery under spinal anesthesia.

## Background

Orthopedic surgery is a surgical procedure used to treat musculoskeletal problems that affect the bones, joints, muscles, tendons, and ligaments caused by accidents, trauma, injury, or chronic conditions [1, 2]. The most common form of neuraxial technique used in orthopedic surgery is spinal anesthesia due to its excellent intraoperative pain control, decreased blood loss, and postoperative analgesia [3, 4]. Even if spinal anesthesia is commonly preferred, post-spinal shivering (PSS) is one potential complication of spinal anesthesia that has a high incidence in orthopedic surgery in developing countries due to a lack of facilities to maintain normothermia [5, 6]. It has been reported to be 8.15% in sub-Saharan tertiary hospitals and 11.6% in Ethiopia [7, 8]. The high incidence of PSS is related to hypothermia, which occurs during the intraoperative period because of vasodilatation and loss of thermoregulatory vasoconstriction below the level of spinal block, which results in heat redistribution from the core to the peripheral body [9]. Studies show that the incidence is also determined by other factors like the type of anesthesia used, age and gender of the patient, duration of anesthesia and surgery, type of surgery, and orthopedic surgery [10].

Untreated PSS can lead to serious complications such as exacerbation of wound pain, delayed wound healing, increased metabolic demand, increased oxygen consumption, and hemostatic dysfunction, especially in patients with low cardiac reserve and arterial hypoxia [11]. PSS has been treated and prevented in a variety of ways. To reduce the occurrence of the condition, non-medical methods such as reflective blankets, cutaneous forced-air warming devices, warm humidified anesthetic gases, and radiant heat are used. This equipment was effective in maintaining normal body temperature, but it was costly and not practical in all settings [12]. Furthermore, it is more logical to prevent the problem and maintain normothermia during neuraxial anesthesia than to treat it once it has occurred [13, 14]. Medical methods are the most common and cost-effective approach in clinical practice.

Different literature shows the efficacy of anti-shivering medications like clonidine, meperidine, tramadol, nefopam, hydrocortisone, dexmedetomidine, and ketamine were the best performing pharmacological agents [15–17]. Most of these drugs are effective in the prevention of PSS, but they have various side effects [18, 19], so that a study mentions two drugs, ketamine and tramadol. Ketamine is a noncompetitive N-methyl-D-aspartate (NMDA) receptor antagonist, with a role in thermoregulation secondary to inhibition of norepinephrine uptake, and can reduce heat redistribution from the core to the periphery [20]. In addition to being a noncompetitive NMDA antagonist, it has several other pharmacological properties, like an opioid agonist, blocking amine uptake in the descending inhibitory monoaminergic pain pathway, having a local anesthetic action, and interacting with muscarinic receptors [21].

Tramadol is an atypical centrally acting weak opioid that acts at multiple sites. It has a modulatory effect on central monoaminergic pathways, inhibiting the neuronal uptake of noradrenaline and serotonin in the spinal cord and increasing hydroxyl tryptamine (HT) secretion, which resets the body's thermoregulatory center. Intravenous tramadol has a well-established anti-shivering effect with fewer side effects, offers rapid onset, less recurrence, and low cost, is easily available in the operating room, and is easy to implement when compared with other opioids [22, 23].

In the study setting, low-dose ketamine and tramadol have usually been used for prophylactic control of post-spinal anesthesia shivering. However, various studies have shown contradicting views on the effectiveness of prophylactic low-dose ketamine and tramadol [6, 24]. There is also a gap in showing better prophylactic agents concerning the effectiveness of prevention of PSS incidence, severity, and occurrence of adverse effects [25, 26]. Furthermore, updating health professionals with new and alternative methods to prevent post-spinal shivering during surgery is a necessary option for evidence-based clinical practice. Hence, this study is intended to compare the effectiveness of prophylactic intravenous 0.25 mg/kg ketamine versus 0.5 mg/kg tramadol in the prevention of PSS in those undergoing orthopedic surgery under spinal anesthesia at the comprehensive specialized hospital of Wolaita Sodo University.

## Methodology

### Study design and patients

An institutional-based prospective cohort study was conducted at the Wolaita Sodo University comprehensive specialized hospital in Sodo town, the administrative center of Wolaita Zone, which is 339 km away from Addis Ababa, the capital city of Ethiopia. The study was carried out following receipt of an ethical clearance letter from the Institutional Review board of Wolaita Sodo University, College of Health Science and Medicine. Before beginning to collect data, the letter was given to each administrative body at the hospital to obtain their informed consent. Each patient gave written, informed consent after being told of the benefits and goals of the study. Confidentiality was maintained at all levels of the study.

All adult patients who underwent surgery at the comprehensive specialty hospital of Wolaita Sodo University between February 28 and August 28, 2020, served as the population's source, and the study subjects were chosen from among adult elective patients who underwent orthopedic surgeries under spinal anesthesia during the study period. Those patients who developed shivering before spinal anesthesia, hypotensive or hypovolemic patients, patients who needed a blood transfusion during the study period, patients who received vasodilation agents before spinal anesthesia, patients who were premedicated by opioid analgesia, patients who received pethidine for treatment of shivering, patients who took ketamine or tramadol other than the study dose, and patients who took a second dose of ketamine or tramadol other than the study dose were excluded from the study. The study included men and women between the ages of 18 and 60, as well as ASA class I-II patients who underwent orthopedic surgery under spial anesthesia. In the study setting, pethidine, tramadol, and ketamine were used as medications for post-spinal shivering management during orthopedic surgery. The patients were not randomized since RCT is not allowed at our university. Instead, patients were grouped into those who received an intravenous prophylactic dose of Ketamine before administration of spinal anesthesia for orthopedic surgery, called the K group (*n* = 258), and patients who received Tramadol were grouped as the T group (*n* = 258) based on the responsible anesthetists' decision, and the patients who took T for the prevention of PSS are considered the control groups.

### Sample size calculation

Based on the following assumptions, the sample size was calculated using the double population proportion formula for comparing two proportions:—significance level 5% (α = 0.05), power of the study (1 – β) of 80%, from the previous study, tramadol and ketamine were found to prevent post-operative shivering by 41.5% and 53.7%, respectively [18]. It has been computed as follows:

$$\mathrm{n}1=\mathrm{n}2= \frac{\mathrm{P}1 \left(1-\mathrm{P}1\right)+\mathrm{P}2 \left(1-\mathrm{P}2\right)\times {\left(\mathrm{Z}+\upbeta \right)}^{2}}{{\left(\mathrm{P}1 -\mathrm{ P}2\right)}^{2}}$$

where:

n1 = number of clients to take ketamine.

n2 = number of clients to take tramadol.

Z = 95% confidence interval = 1.96.

F (α, β) = the power function at 80% = 0.84.

P1 = Reduce incidence of shivering ketamine group is 1-P1 = 0.585.

P2 = Reduce incidence of shivering tramadol group is 1-P2 = 0.463.

n1 = n2 = 258, total sample size for both group is 516. Following the lottery selection of the first random case, patients who met the inclusion criteria were consecutively selected into the study with the assumption that the study participant by itself was randomly admitted and the procedure continued till the required sample size was met in each group.

### Data collection tools and procedure

Data was collected from 516 participants using an interviewer-administered questionnaire and checklist. Three BSc anesthetists were used for data collection and one master’s holder anesthetist supervised them. Before recruiting patients into the study, training and orientation about the objective and process of data collection were provided by the principal investigator. At the pre-anesthetic visit, patients were informed regarding the study protocol and obtained informed written consent from all patients before including them in the study and keeping confidentiality. Patient monitoring devices such as pulse oximeters, noninvasive blood pressure, axillary thermometers, and electrocardiography were attached to the patients, and the hemodynamic parameters like oxygen saturation, blood pressure, pulse rate, respiratory rate, and temperature were monitored throughout the procedure. Before spinal anesthesia, all patients were preloaded with normal saline at 10 mL/kg to prevent spinal anesthetic-induced hypotension.

In the sitting position, with a strict aseptic approach at L3-L4 or L4-L5, spinal anesthesia was delivered with 22–25-gauge Quincke spinal needles and 3 ml of 0.25 percent Bupivacaine. For the prevention of post-spinal shivering, before spinal anesthesia and after the hemodynamic stability was checked, an intravenous prophylactic dose of ketamine 0.25 mg/kg or tramadol 0.5 mg/kg was provided based on the preference of the anesthesia provider. The shivering scale was measured throughout surgery at 10-min intervals up to 60 min. Also, for 60 min, axillary body temperature was monitored and recorded using a standard non-invasive monitor at 10-min intervals. Intraoperatively, the adverse effects like nausea, vomiting, sedation, and hypotension were also passively followed and recorded every 10 min for 60 min. The intra-operative variables were filled out by the anesthetist in charge, and the other postoperative variables were collected by other trained data collectors. Under careful supervision, data consistency and completeness were checked throughout the data collection and data entry.

### Data processing and analysis

The data was then manually checked for completeness and coded, then entered into Epi Data 3.14 computer software by the investigator and exported to SPSS version 23 computer software for analysis. The normality distribution of the data was checked by the Shapiro–Wilk test. Continuous data was compared using a student’s t-test and a chi-square test was used to compare categorical data. Bivariate and multivariate analysis were used to check the association of each variable with post-spinal shivering. To control for the possible effect of confounding, a variable that had a *P*-value of 0.25 in the bi-variate analysis was entered into the multivariable logistic regression model. In the multivariable logistic regression, the variables that had a significant correlation with a *p*-value lower than 0.05 were regarded as independent factors. To present the findings, tables, charts, and graphs were used.

### Operational definition

**Effectiveness:** is measured by a lower incidence of post-spinal shivering, a lower severity of post-spinal shivering, and a lower number of adverse effects following the induction of prophylactic agents for orthopedic surgery within 60 min.

**The incidence of shivering:** is the number of shivering events that occur in the operation room after the administration of low dose ketamine or tramadol for the prevention of post-spinal shivering.

**Low-dose ketamine:** a dose of 0.25 mg/kg ketamine used for the prevention of shivering after spinal anesthesia.

**Prophylactic:** is an administration of intravenous ketamine or tramadol before shivering starts or soon after spinal anesthesia.

**Post-spinal shivering control**: a patient who was induced with prophylactic agent ketamine or tramadol and post-spinal shivering severity scale is < 2 within 60 mints.

The incidence of post-spinal shivering was graded using a scaled grade. 0 = no shivering, 1 = piloerection but no visible shivering, 2 = muscular activity in only one group, 3 = muscular activity in more than one muscle group but not generalized, and 4 = shivering involving the whole body [27].

**Hypotension:** defined as a decline in mean arterial blood pressure (MAP) of more than 20% from the baseline.

The Ramsey sedation score was used to assess intraoperative sedation level. The degree of sedation was assessed as 1 = fully awake and oriented, 2 = drowsy, 3 = eyes closed but arousable to command, 4 = eyes closed but arousable to mild physical stimulation, 5 = eyes closed but sluggish response to mild physical stimulation or audible command, and 6 = no response to painful physical or loud auditory stimulus [28]

## Results

### Socio-demographic and preoperative characteristics

A total of 516 patents were involved in the study, with a response rate of 100%. Among the participants, 303 (58.7%) were 18–35 years old, and the majority of study participants in both groups were male, 376 (72.8%). The mean weight (kg) of patients in the ketamine and tramadol groups was 62.6 ± 18.67 and 62.1 ± 18.49, respectively, with a *p*-value of 0.44. Only a few were ASA II patients in both groups (Table 1).

**Table 1:** Socio-demographic and preoperative characteristics of patients who underwent orthopedic surgery at the comprehensive specialized hospital of Wolaita Sodo University from February 28/2020–August 28, 2020

Variable	Category	Ketamine group (258)	Tramadol group (258)	Total	*P*-value
Age	18–35 years	163(63.2%)	140(54.3%)	303(58.7%)	0.21
	36–55 years	78(30.2%)	88(34.1%)	166(32.17%)	
	> 55 years	17(6.6%)	30(11.6%)	47(9.1%)	
Gender	Male	177(68.6%)	199(77.1%)	376(72.8%)	0.15
	Female	81(31.4%)	59(22.9%)	140(27.1%)	
Ethnicity	Wolaita	96(37.1%)	83(32.2%)	179(34.7%)	1.92
	Amara	19(7.1%)	14(5.4%)	33(6.4%)	
	Oromo	38(14.7%)	45(17.4%)	83(16.08%)	
	Gurage	17(6.6%)	39(15.1%)	56(10.8%)	
	Hadia	27(10.5%)	19(7.4%)	46(8.9%)	
	Gamo	32(12.4%)	25(9.7%)	57(11.05%)	
	Sidama	10(3.9%)	16(6.2%)	26(5.04%)	
	Others	17(6.6%)	16(6.6%)	33(6.4%)	
Residency	Urban	191(74.0%)	192(74.4%)	383(74.2%)	0.16
	Rural	67(26.0%)	66(25.6%)	133(25.7%)	
ASA status	ASA I	248(96.1%)	231(89.5%)	479(92.83%)	0.21
	ASA II	10(3.9%)	27(10.5%)	37(7.17%)	
Religion	Orthodox	83(32.2%)	95(36.8%)	178(34.5%)	0.06
	Protestant	121(46.9%)	103(39.9%)	224(43.4%)	
	Muslim	40(15.5%)	50(19.4%)	90(17.4%)	
	Catholic	13(5.0%)	11(4.3%)	24(4.6%)	
Weight		62.6 ± 18.67	62.6 ± 18.67		0.44

Hint: n (%) = Number and percentage, mean (standard deviation) = mean ± SD. The value is given as the mean ± SD for weight, and number of patients or frequency for the rest

### Intraoperative hemodynamic parameters

The independent t-test revealed that the mean score of preoperative baseline MAP and HR in the ketamine group was 93.28 ± 6.61 and 81.41 ± 10.02, as well as the mean score in the tramadol group was 95.55 ± 7.35 of MAP and 80.89 ± 9.48 of heart rate. And the intraoperative hemodynamic parameters had a statistically significant difference in both groups after 20 min with a *p*-value of less than 0.001 in MAP (Table 2).

**Table 2:** Intraoperative hemodynamic parameters in patients who underwent orthopedic surgery at the comprehensive specialized hospital of Wolaita Sodo University from February 28/2020–August 28, 2020

Hemodynamic parameter	Ketamine group(*n* = 258)	Tramadol group(*n* = 258)	t-test	*P*-value
Baseline MAP(Mean ± SD)	93.28 ± 6.61	95.55 ± 7.35	3.690	0.061
After 10mints	86.62 ± 5.03	86.33 ± 6.18	0.594	0.553
After 20mints	83.75 ± 4.46	80.40 ± 5.51	7.611	0.001
After 30mints	83.41 ± 4.23	79.07 ± 5.14	10.469	0.001
After 40mints	83.34 ± 4.25	79.28 ± 4.62	10.405	0.001
After 50mints	83.64 ± 4.47	79.51 ± 4.88	10.038	0.001
After 60mints	83.91 ± 4.18	80.00 ± 4.71	9.965	0.001
Baseline HR(Mean ± SD)	81.41 ± 10.02	80.89 ± 9.48	0.605	0.546
After 10mints	80.45 ± 10.00	78.49 ± 10.74	2.146	0.032
After 20mints	80.22 ± 10.66	76.97 ± 10.84	3.440	0.001
After 30mints	80.42 ± 10.90	77.11 ± 10.57	3.502	0.001
After 40mints	81.09 ± 10.84	78.52 ± 10.94	2.688	0.007
After 50mints	82.52 ± 10.43	80.84 ± 12.68	1.646	0.100
After 60mints	85.59 ± 11.88	85.20 ± 13.48	0.350	0.727

*n* Number of patients, *BPM* Hart beat per-minutes, data presented as mean ± SD, *P*-value < 0.05 considered as significant

**Fig. 1:**
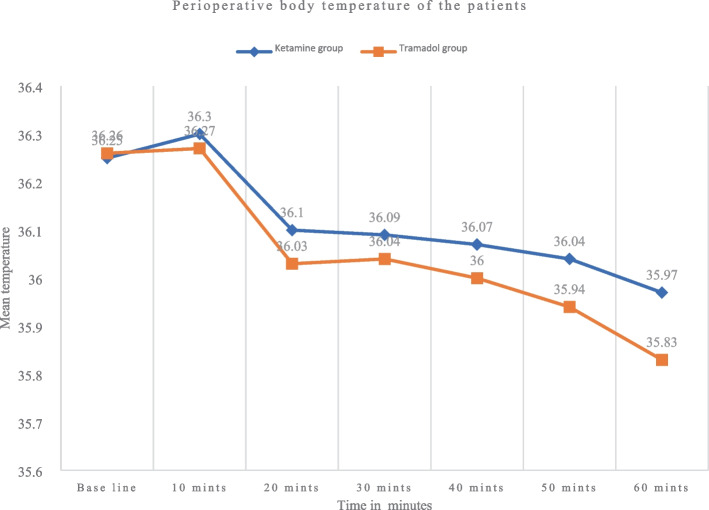
Axillary body temperature during intraoperative period in those patients undergoing orthopedic surgery at the comprehensive specialized hospital of Wolaita Sodo University from February 28/2020–August 28, 2020

There was a statistically significant difference in body temperature between the two groups, with *p*-values of 0.003, 0.001, and 0.001 at a time interval of 40, 50, and 60 min, respectively. Patients in the tramadol groups had decreased body temperature from baseline values after spinal anesthesia (Fig. 1).

The mean (± SD) intraoperative intravenous fluid used was 987.29 ± 222.15 and the duration of prophylactive agent was 54.15 ± 7.96 in the ketamine group, and whereas 1032.17 ± 460.11 and 51.32 ± 8.59 in the tramadol group (Table 3).

**Table 3:** Intraoperative patient status and duration of prophylactic agent in patients who underwent orthopedic surgery at the comprehensive specialized hospital of Wolaita Sodo University from February 28/2020–August 28, 2020

Characteristic	Ketamine group (*n* = 258)	Tramadol group (*n* = 258)	*P*-value
Total intravenous fluid used in ml (mean ± SD)	987.29 ± 222.15	1032.17 ± 460.11	0.159
Blood loss during surgery in ml (mean ± SD)	103.28 ± 62.73	108.43 ± 60.50	0.342
Duration of prophylactive agent in minutes (mean ± SD)	54.15 ± 7.96	51.32 ± 8.59	0.001

*n* Number of patients, *ml* Milliliter, data presented as mean ± SD, *P*-value < 0.05 considered as significant

### The Incidence and severity of shivering

**Fig. 2:**
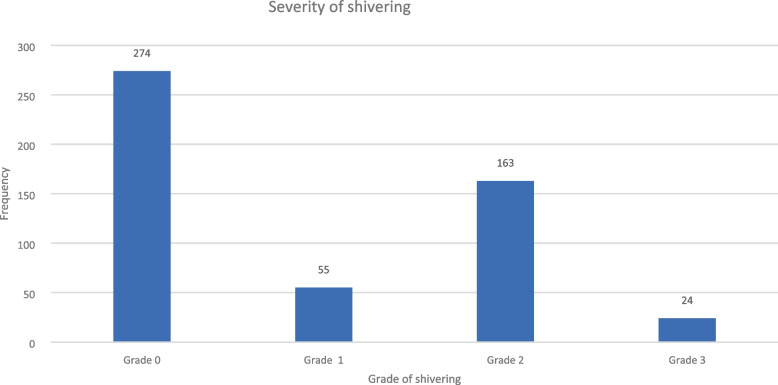
Severity of shivering in those patients undergoing orthopedic surgery at the comprehensive specialized hospital of Wolaita Sodo University from February 28/2020–August 28, 2020

**Fig. 3:**
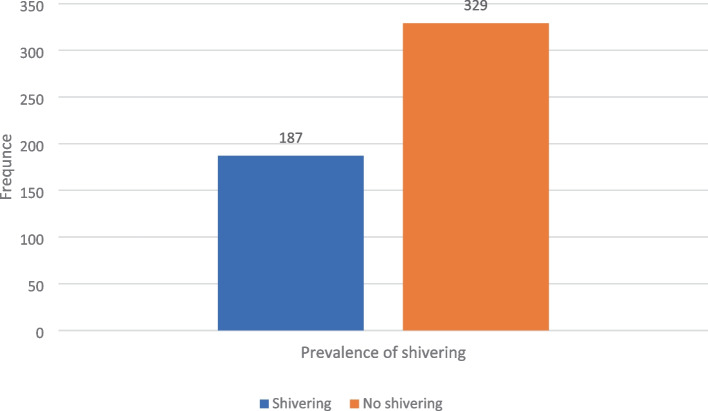
Distribution of shivering in those patients undergoing orthopedic surgery at the comprehensive specialized hospital of Wolaita Sodo University from February 28/2020–August 28, 2020

The overall incidence of post-spinal shivering was 187 out of 516 (36.2%) and a significant difference was observed regarding the incidence of shivering in both groups with a *p*-value of 0.002. The severity of shivering proportion in grades two and three was much higher in tramadol than in the ketamine group (Table 4) and (Fig. 2), and the over-all distribution of post-spinal shivering was high in the tramadol group (Fig. 3).

**Table 4:** Incidence of shivering in those patients who underwent orthopedic surgery at the comprehensive specialized hospital of Wolaita Sodo University from February 28/2020–August 28, 2020

Groups	Incidence of shivering		Chia square	
	**Yes**	**No**	**X**^**2**^	***P*-value**
Ketamine group (%)	74(28.7%)	184(71.3%)	12.70	0.002
Tramadol group (%)	113(43.8%)	145(56.2%)		

Data presented as number of patients, percentage and relative risk, *P*-value < 0.05 considered as significant

### Adverse effects of the prophylactic agents

The incidence of nausea and vomiting was statistically highly significant in tramadol group 157 (60.9%) with a *p*-value of 0.001. And the intraoperative sedation was more marked in the ketamine group compared to the tramadol group, with a *p*-value of 0.001 (Table 5).

**Table 5:** Adverse effect of a prophylactive agent in those patients who underwent orthopedic surgery at the comprehensive specialized hospital of Wolaita Sodo University from February 28/2020–August 28, 2020

**Variables**	**Ketamine group (*n* = 258)**	**Tramadol group (*n* = 258)**	**Chi-square**	
			**X**^**2**^	***P*-value**
Nausea and vomiting				
Yes	8(3.1%)	157(60.9%)	197.8	0.001
No	250(96.9%)	101(39.1%)		
Sedation (Ramsay score ≤ 3)				
Yes	104(40.3%)	16(6.2%)	83.8	0.001
No	154(59.7%)	242(93.8%)		

*n* Number of patients, data presented as number of patients and percentage, *P*-value < 0.05 considered as significant

### Factor affecting post-spinal shivering

The results showed that age, ethnicity, baseline body temperature, ASA classification, prophylactic agent, duration of surgery and anesthesia, sedation, and blood loss during surgery were statistically significant in a bivariate analysis at a *p*-value less than 0.25, therefore, included in the multivariate analysis. It was observed as a result of multivariate analysis that age, prophylactive agent, and duration of surgery were strongly associated with post-spinal shivering at a *p*-value less than 0.05.

In this study, low-dose ketamine had a 57.3% protective effect against developing post-spinal shivering (PSS), with an AOR of 0.427 (0.28–0.63). Another significant variable associated with PSS was the duration of surgery and anesthesia. When the duration of surgery increases (AOR 1.47 (1.57–1.58), patients who received either low-dose ketamine or tramadol had a similar risk of developing PSS. In addition, 99.2% of young aged adults and 76% of middle-aged adults were at higher risk of developing PSS compared to older adults with an AOR of 0.08 (0.02, 0.27) and 0.24 (0.07, 0.81), respectively (Table 6).

**Table 6:** Factors associated with post-spinal shivering in those patients who took prophylactic dose of ketamine or tramadol and underwent orthopedic surgery at the comprehensive specialized hospital of Wolaita Sodo University from February 28/2020–August 28, 2020

Variables	Post-spinal shivering		COR (95% CI)	AOR (95% CI)
	**Yes (%)**	**No (%)**		
**Age**				
18–35(young aged adult)	141(46.4)	163(53.6)	0.11(0.04, 0.32)	**0.08(0.02, 0.27)** ***
36–55 (middle aged adult)	42(25.1)	125(74.9)	0.29(0.10, 0.86)	**0.24(0.07, 0.81)** *
> 55 years (Older adults)	4(8.9)	41(91.1)	1	1
**Religion**				
Orthodox	63(32.8)	129(67.2)	1.80(0.53, 1.21)	1.95(0.70, 4.92)
Protestant	83(39.3)	128(60.7)	1.42(0.60, 1.74)	1.38(0.48, 3.34)
Muslim	30(33.3)	60(66.7)	0.55(0.23, 1.33)	1.96(0.60, 5.54)
Catholic	11(47.8)	12(52.2)	1	1
**Ethnicity**				
Wolaita	70(39.1)	109(60.9)	0,83(0.39, 1.76)	0.69(0.29, 1.64)
Amhara	14(42.4)	19(57.6)	1.08(0.63, 1.85)	0.59(0.20, 1.75)
Oromo	29(34.9)	54(65.1)	1.02(0.55, 1.89)	0.60(0.23, 1.58)
Gurage	21(37.5)	35(62.5)	1.73(0.84, 3.58)	0.70(0.25, 1.92)
Hadia	12(26.1)	34(73.9)	1.13(0.60, 2.11)	1.21(0.41, 3.48)
Gamo	19(33.3)	38(66.7)	0.71(0.31, 1.63)	0.75(0.28, 2.01)
Sidama	12(46.2)	14(53.8)	1.39(0.64, 3.00)	0.50(0.15, 1.62)
Others	10(27.8)	26(72.2)	1	1
**ASA classification**				
ASA I	185(37.2)	312(62.8)	0.197(0.45,0.861)	0.47(0.09, 2.51)
ASA II	2(10.5)	17(89.5)	1	1
**Body temperature**				
< 36.0	65(37.6)	108(62.4)	0.98(0.67, 1.44)	0.24(0.07, 0.80)
36.1–36.9	118(37.1)	200(62.9)	0.32(0.10, 0.96)	0.40(0.12, 1.29)
> 37.0	4(16.0)	21(84.0)	1	1
**Prophylactive agent**				
Ketamine	74(28.7)	184(71.3)	0.50(0.35–0.73)	0.427(0.28, 0.63) ***
Tramadol	113(45.8)	145(56.2)	1	1
**Duration of surgery**				
≤ 1 h	177(68.9)	80(31.1)	1	1
> 1 h	11(4.2)	248(95.8)	17.66(7.2–43.34)	1.47(1.37, 1.58) ***
**Blood loss during surgery**				
< 100 ml	91(39.7)	138(60.3)	1.63(1.09, 2.42)	1.47(0.80, 2.68)
100–199 ml	62(28.7)	154(71.3)	0.77(0.45, 1.32)	2.36(1.28, 4.34)
≥ 200 ml	34(47.9)	37(52.1)	1	1
**Sedation**				
Yes	31(28.2)		1.597(1.01–2.532)	0.81(0.47, 1.37)
No	155(38.3)	79(71.8) 250(61.7)	1	1

Data presented as number of patients and percentage

*COR* Crud odd ratio, *AOR* Adjusted odd ratio

^*^p-value < 0.05

^***^p-value < 0.001

## Discussion

Post-spinal shivering is a common problem during anesthesia and surgery. Thus, the prevention of post-anesthesia shivering is relevant clinical practice. Ketamine and tramadol are among the prophylactic agents used for intraoperative shivering. Different evidence depicts the effectiveness of pharmacological drugs like ketamine, pethidine, and tramadol in preventing post-spinal shivering. Pethidine has a better outcome in preventing PSS but is mostly associated with adverse events like respiratory depression and arterial oxygen desaturation, nausea, vomiting, and sedation in some patients [19, 23, 29]. Low-dose ketamine and tramadol, on the other hand, have fewer or no adverse effects like mild sedation and hallucinations [17, 30]. However, certain studies have shown contradictory views on the effectiveness of prophylactic low-dose ketamine and tramadol in preventing post-spinal shivering [6]. Furthermore, despite the fact that high doses of ketamine and tramadol were effective in controlling post-spinal shivering, their side effects limited their use [31, 32]. In our study, the effectiveness of low-dose ketamine versus tramadol in reducing intraoperative shivering among orthopedic surgical patients under spinal anesthesia was compared.

The overall incidence of post-spinal shivering in the current study was 36.2%. That is higher than the study done in North West Ethiopia, with a prevalence of 25.6% [7]. Our finding is much higher than studies conducted elsewhere, with a range of 8–14.4% [10, 33]. On the other hand, our finding is lower than the study conducted in Khyber Teaching Hospital, Pakistan. The difference could be due to pre-warmed intravenous fluid (IV) and controlling the operation room temperature between 24 and 26 oC [34].

In the current study, the incidence of post-spinal shivering was significantly lower among patients taking low-dose ketamine as compared to the tramadol group. This finding was corroborated with a study conducted on the effectiveness of ketamine in Abubakar Tafawa Balewa Teaching Hospital, Bauchi, Nigeria [35]. In contrast to this, no significant difference was observed between the two prophylactic drugs, according to the study conducted in India. This could be due to PSS being recorded for only 30 min after the surgery, where prolonged follow-up is used in the current study [36].

The incidence of intraoperative shivering was 28.7% in the ketamine group and 43.8% in the tramadol group. This is in line with the study conducted in a combined military hospital and medical college in Lahore, Pakistan, which revealed that the incidence of intraoperative shivering was 18.75% in the ketamine group and 46.88% in the tramadol group [37]. Our study result is lower than the study finding reported in Gondar, with an incidence of 41.5% and 53.7% among ketamine and tramadol groups, respectively [18]. In contrast, the study conducted in Jinnah Hospital Lahore showed that the incidence of shivering was 6% in the tramadol group and 32% in the ketamine group, which may be due to the infusion of the prophylactive agent and pre-warmed intravenous fluid used for the load [38].

In the current study, the severity of shivering scores 1, 2, and 3 was higher in the tramadol group than in the ketamine group. This is in line with the study conducted in Mustasharak hospital, Egypt [39]. Our result shows that the severity of shivering in the ketamine group was 27.9% in score 2, whereas in the tramadol group it was 35.3%. This finding is comparable with the 22% and 31.7% documented in Gondar and the study findings reported in Indonesia, with a severity score in ketamine of 23.3% whereas in the tramadol group it is 26.7%. The difference between our study and the previous one could be due to more than one-hour of follow-up in the current study and the study that included the high block. According to the study conducted in India, the severity of shivering was higher in the ketamine group than in the tramadol group. This could be due to premedication of Midazolam and Fentanyl [40].

Concerning grade 3 score shivering in our study, it is less than 1% in ketamine and 8.5% in tramadol. This finding is much lower than the finding in North West Ethiopia, where shivering scores greater than 19% are reported after both drugs. This could be due to the small sample size and a pregnant mother with an age less than 39 years [18].

In the present study, there was a significant change in intraoperative hemodynamic parameters between the two groups, and mean arterial blood pressure was higher in the ketamine group. This result is in line with a comparative study conducted in India which showed that mean arterial blood pressure was higher in patients who received ketamine as compared to the placebo group [17]. According to the studies documented in India, intraoperative hemodynamic parameters in the ketamine and tramadol groups did not show significant changes in hemodynamic parameters [41] This could possibly be due to a preload with pre-warmed IV fluid to 37 oC and ketamine being a sympathomimetic agent that increases the mean arterial blood pressure.

Our study results showed the incidence of intraoperative nausea and vomiting was significantly higher in the tramadol group when compared to the ketamine group, which is in line with a comparative study conducted in Nishutar Medical College and Hospital, Multan, which reveals the incidence of nausea and vomiting is low in the ketamine group [42]. In another study that was conducted in Calcutta National Medical College and Hospital, India, there was an increased incidence of nausea and vomiting in the tramadol and pethidine groups [43]. In contrast, the study conducted in Songklanagarind hospital, Thailand reveals that there is no statistically significant difference in the incidence of nausea and vomiting in between ketamine and placebo groups, which may be due to general anesthesia started by giving propofol and narcotics [6].

The incidence of low levels of sedation was higher in the ketamine group when compared to the tramadol group. One comparative study also investigated the incidence of sedation scores and found that it was significantly higher in the ketamine group as compared with the tramadol group [16]. In contrast, the study conducted at Siddhardha Medical College, India revealed that sedation scores of ketamine and tramadol were statistically significantly higher than those of dexamethasone. This may be due to premedication of Midazolam and Fentanyl [40].

In the previous study conducted in Safdarjung hospital, India, there was a greater fall in core temperature in the placebo group as compared with the ketamine, tramadol, and clonidine groups. In our study, there was a significant decrease in mean temperatures after spinal anesthesia with respect to baseline value and changes over time in the ketamine and tramadol groups. This result is in line with a study conducted in the Faculty of Medicine et al.-Azhar University, Egypt [39]. Another study conducted in the institute civil hospital, Aizawl, Mizoram, showed that the decrease in core temperature was statistically significant in ketamine compared to the baseline level. This could be due to the vasodilation effect of spinal anesthetic agents [44]. In contrast, the study conducted in the faculty of medicine, Tanta University, Egypt, showed the change in the mean temperature in the tramadol group was not statistically significant at any time of the post-anesthesia period. That may be because they measured tympanic membrane temperature [45].

The result of our study shows that the aged group has a strong association with a reduced risk of post-spinal shivering, which is in line with a study conducted at the University of Gondar, in 2015 [7]. That may be due to diminished thermoregulatory response to changes in body temperature in old age or it could be due to the atrophy of skeletal muscles [10].

Our study results showed that those patients with a duration of surgery greater than 1 h were strongly associated with post-spinal shivering, which is in line with a comparative study conducted at the University of Gondar, Ethiopia [7]. However, according to the study conducted at the University of Marburg, Germany in 2005, there was no strong association. This could be because of the general anesthesia and the patient's assessment at the post-anesthesia care unit [10].

Our study results showed that patients who had taken low-dose ketamine had a statistically significant association with the prevention of PSS. This finding is supported by similar studies conducted in Pakistan, Turkey, and Iran [26, 46, 47].

In this study, hypothermia was not associated with post-spinal shivering. In contrast to our finding, a study conducted in Gondar showed that hypothermia was associated with PSS [7]. This may be due to the type of anesthesia used during surgery.

### Limitation

The limitation of this study was the failure to measure core temperature, which is the more accurate way to assess intraoperative temperature.

### Strength

Patients were followed for one hour during the surgery to observe the incidence of shivering, which is higher than most of the literature in the country and elsewhere.

## Conclusion

The magnitude of shivering in this study was 28.7% in the ketamine group and 43.8% in the tramadol group. Low-dose ketamine showed a better outcome than tramadol in reducing the frequency and incidence of shivering after spinal anesthesia. The age groups of 18–35 years and 36–55 years, as well as the length of surgery, were found to be predictors of post-spinal shivering. In addition, low-dose ketamine restored hemodynamics with a low incidence of intraoperative nausea and vomiting when compared to tramadol.

Even though low-dose ketamine and tramadol prophylaxis can prevent post-spinal shivering for patients undergoing orthopedic surgery under spinal anesthesia, low-dose ketamine is more effective and available than tramadol. Therefore, it is recommended that low-dose ketamine be effective for the prevention of PSS. A randomized controlled trial should be done by measuring core temperature to limit the confounding factors.

## Data Availability

All data and materials in this manuscript are available from the corresponding author on reasonable request.

## Abbreviations

ASA: American Society of Anesthesiology
AOR: Adjusted Odds Ration
BP: Blood Pressure
HR: Heart Rate
HT: Hydroxy Tryptamine
IV: Intravenous
MAP: Mean Arterial Pressure
NMDA: N-methyl D-aspartate
OR: Operation Room
PACU: Post-Anesthesia Care Unit
PAS: Post-Anesthesia Shivering
PI: Principal Investigator
PSS: Post-Spinal Shivering
RCT: Randomized Control Trial
SA: Spinal Anesthesia
SD: Standard Deviation
SPSS: Statistical Package for Social Science

## Declarations

### Competing of interests

The authors declare that they have no conflicts interests.
